# Family Care, Economic Stress, and Depressive Symptoms Among Chinese Adults During the COVID-19 Outbreak: Difference by Urban and Rural Areas

**DOI:** 10.3389/fpsyt.2021.700493

**Published:** 2021-07-21

**Authors:** Chengbin Liu, Liyuan Zhang, Farooq Ahmed, Muhammad Shahid, Xiaohua Wang, Yiqing Wang, Junlan Wang, Jing Guo

**Affiliations:** ^1^School of Sociology, Huazhong University of Science and Technology, Wuhan, China; ^2^Nutritional Anthropologist, Department of Anthropology, Quaid-e-Azam University, Islamabad, Pakistan; ^3^Former Research Associate University of Washington, Seattle, WA, United States; ^4^World Health Organization, Balochistan, Pakistan; ^5^School of Social Development and Public Policy, Beijing Normal University, Beijing, China; ^6^Department of Psychology, University of York, York, United Kingdom; ^7^Department of Health Policy and Management, School of Public Health, Peking University, Beijing, China

**Keywords:** family care, depressive symptoms, COVID-19, economic stress, urban-rural difference

## Abstract

**Background:** Although several studies have shown an association of family care with a high level of depressive symptoms, the relationship between them remains indistinguishable.

**Objective:** This study aims to examine the associations between family care, economic stress, and depressive symptoms among Chinese adults in urban and rural areas during the COVID-19 outbreak.

**Methods:** Based on cross-sectional data collected through online surveys from February 1st to 10th 2020 in China the present study recruited 2,858 adults. It used multiple linear regression to examine the association between family care and depressive symptoms, while economic stress was examined as moderators on the above relationship.

**Results:** The results showed that caring for both the elderly and children was significantly associated with higher depressive symptoms compared with non-caregivers (*B* = 2.584, 95%CI: 1.254, 3.915), and a similar result was also found in urban areas. Also, caring for the elderly only was also had a higher level of depressive symptoms than non-caregivers in rural areas (*B* = 3.135, 95%CI: 0.745, 5.525). Meeting the care needs was significantly associated with lower depressive symptoms compared with unmet care needs, while for rural caregivers, the results were not significant. Besides, economic stress strengthened the effect of family care needs on depressive symptoms for sandwich-generation caregivers who provide care to both the elderly and children (*B* = 0.605, 95%CI: 0.077, 1.134). While in rural areas, the moderation effects of economic stress were only found for elderly caregivers (*B* = 1.106, 95%CI: 0.178, 2.035).

**Conclusion:** These findings suggest that we should pay more attention to the family caregiver's mental health during the COVID-19 outbreak. In addition, more effective policies should be developed to provide financial support for family caregivers, especially for sandwich-generation caregivers and rural elderly caregivers.

## Introduction

Mental health problems, such as depressive symptoms might have been triggered by public health crises and growing financial loss amid and after the COVID-19 outbreak (1, 2). The family care environment has also been affected during the COVID-19 outbreak. On the one hand, the outbreak of COVID-19 posed a challenge to the health care system, informal family caregivers had to provide complex care to family members with illness (3). On the other hand, most of the operations in educational institutions and welfare facilities were shut down under lockdown policy (4, 5), and more tasks regarding care were reassigned to the family, which increased the extra burden of family caregivers. As a consequence, the social disruption caused by the COVID-19 pandemic augmented psychological distress among family caregivers (6). Furthermore, owing to several factors, such as the traditional culture of filial piety, lack of social care resources, etc., the family still bore the primary responsibility for care in China (7, 8). For example, one study showed that 92.71% of family members provided elderly care (9). At the same time, because of the lack of formal care, child care was mainly undertaken by family members such as parents and grandparents (10). In conclusion, unlike developed countries, in China, due to the lack of a developed social care system, family care was almost entirely dependent on the family members (8). As a result, the limited access to healthcare resources and the closure of public services put more care pressure on family caregivers who already had a heavy care task. Therefore, there is a need to focus on the mental health of family caregivers during the epidemic, especially in China.

### Family Care and Mental Health Symptoms

Family care generally refers to family caregivers assisting family members who need care, usually unpaid (11), which involves care needs and satisfaction in our study. Previous studies have analyzed the relationship between family care and depressive symptoms, but with inconsistent results. Some researchers found that family care needs were related to a high level of depressive symptoms (12–14), while recent research started to focus on the positive outcomes of family caregiving (e.g., satisfaction, self-esteem) (15, 16). Moreover, according to the stress model, meeting the care demands was a stressor for caregivers, which was associated with caregivers' depressive symptoms (17, 18). On the contrary, another study found that caregivers whose care needs were satisfied would be less likely to suffer depressive symptoms, even if they were facing the high stress of caregiving (19). Although studies have emphasized the importance of family care needs and care needs satisfaction during the pandemic context (6, 20, 21), the specific relationship between these factors and depressive symptoms has not been established. Furthermore, none of these studies have examined the relationship between care needs, care needs satisfaction and depressive symptoms simultaneously. Thus, to clarify the relationship between these factors, it is necessary to examine the implicit connection of family care needs, care needs satisfaction, and depressive symptoms during the pandemic among adults in China.

### Comparison of Different Types of Caregivers

In China, childcare played an important role in family care (10). Nonetheless, a large number of studies focused on eldercare instead of childcare and found that family care was associated with depressive symptoms (22, 23). Unlike childcare, the deterioration of physical health among the aged was observed owing to the reason that they need more care (24, 25). Moreover, with the life cycle theory, eldercare elicited more negative emotions, whereas childcare generated more positive emotions (26). These differences often put sandwich-generation caregivers (i.e., caring for both the elderly and the children) in a difficult situation, as they had to meet the care needs of two conflicting roles (27). Additionally, the risk from COVID-19 may vary by the type of caregivers, for example, COVID-19 represented a greater threat to older adults than children (28), and sandwich-generation caregivers were at high risk of infection owing to the multigenerational characteristics of care (29). Surprisingly, most studies compared caregivers as a whole with non-caregivers (12, 30). Thus, there are no studies to examine the relationship between different types of family care and depressive symptoms during the COVID-19 outbreak. Therefore, research is needed on the association of depressive symptoms among sandwich-generation caregivers, caregivers of children only, and caregivers of the elderly only compared with non-caregivers.

### Economic Stress as a Moderating Variable

During the COVID-19 outbreak, economic stress could affect the impact of family care on caregiver depressive symptoms. According to the stress process theory, life events such as the lockdown policy may bring adverse changes to people's family roles, and these stresses would increase their mental health risk (6, 31), and even under normal circumstances, financial problems were associated with a high level of depressive symptoms among family caregivers (32). In the context of COVID-19, acute economic stress such as immediate unemployment and financial insecurity increased the risk of caregivers' mental health symptoms (6). Thus, there is a need to take economic stress into account as an interactive factor that exacerbates family caregivers' depressive symptoms.

### Urban vs. Rural Differences

Considering urban-rural dual structure and epidemic background in China, associations between family care, economic stress, and depressive symptoms during the pandemic may differ between the urban and rural areas. On the one hand, substantial urban-rural disparities emerged in income sources, social welfare, care resources, and the satisfaction of care needs in China (33, 34). As the contradiction between supply and demand intensifies, rural caregivers tended to bear a heavier care burden than urban caregivers (35). Consequently, significant differences were found in depressive symptoms (36). However, all of the above studies were conducted before the COVID-19 outbreak. In addition, although the COVID-19 outbreak had affected both urban and rural areas, the impact of the epidemic was more profound in urban areas because they were closer to the epicenter of the pandemic and had more intensive population movements (37, 38). As a result, the closure of public services and the overburdened medical system in this public health emergency also increased the burden on urban family caregivers (39). Unfortunately, the difference in the relationship between family care and depressive symptoms between urban and rural areas in this challenging period is still unknown. On the other hand, under the dual economic structure, urban-biased social welfare expenditure increased the income inequality between urban and rural areas, making rural and urban caregivers face different economic care pressures (34). During the period of COVID-19, the lockdown policy has increased the financial burden of rural families in particular (40). However, the moderating effects of economic stress on the relationship between family care and depressive symptoms between rural and urban areas have not been examined. Thus, urban-rural differences should be considered when analyzing the relationship between family care, economic stress, and depressive symptoms.

### Objectives and Hypothesis

The objectives of the study include (1) examine the relationship between different types of family care(measured in terms of family care needs and care needs satisfaction) and depressive symptoms, (2) test the moderating role of economic stress on the above relationship, (3) examine the difference of the relationship between family care and depressive symptoms between urban and rural areas, and further investigate rural-urban differences in the moderating effects of economic stress on the relationship between family care and depressive symptoms. Correspondingly, our first hypothesis involves care needs and care needs satisfaction. We hypothesized that the different types of including care needs and care needs satisfaction would be associated with depressive symptoms. In addition, we assume that adult caregivers report higher levels of depressive symptoms compared to adult non-caregivers. Meanwhile, we expect that care needs satisfaction was associated with lower levels of depressive symptoms compared to unmet care needs. Our second hypothesis relates to the moderation effect of economic stress and family care on depressive symptoms. We assume that economic stress may strengthen the relationship between family care and depressive symptoms. Lastly, our hypothesis shows concerns about the difference between rural and urban areas. For family care, we expect that the relationship between family care and depressive symptoms will vary between rural and urban areas. Meanwhile, we assume that the moderating effects of economic stress on the relationship between family care and depressive symptoms differ between rural and urban areas.

## Methods

### Study Design and Participants

Participants were recruited through an online survey called the psychological status of Chinese adults during the COVID-19.This survey was distributed through a web-based platform (https://www.wjx.cn/app/survey.aspx) from February 1st to 10th 2020. Convenience sampling and snowball sampling were used to recruit participants. Initially, several key contact participants in specific groups were selected, for example, a chief nurse, class tutor, or company manager, etc. Next, critical contact participants were requested to distribute the questionnaires to the subjects through their WeChat group (a widely-used communication tool for people in China). Then, the subjects in each WeChat group were asked to send the questionnaire web link to their WeChat friends. The online survey required respondents to answer every question, so there was no missing data in our study. In total, 2,858 valid samples aged over 18 were obtained. It should be noted that non-caregivers were not included in the sample that meets care needs. Thus, only 1,056 samples were included in the model when considered the relationship between care needs satisfaction and depressive symptoms.

### Eligibility Criteria

The inclusion criteria were Chinese citizens who obtained written informed consent and were over 18 years old.

The exclusion criteria were any conditions that affected the quality of the questionnaire, including <10 min of response time, confusion of logic, etc.

### Sample Size

The sample size was calculated using the following formula: N = Z^2^ pq/d^2^ (41). In this sample size calculation, Z = the standard normal deviation [1.96 at 5% of type 1 errors (*P* < 0.05)], *p* = prevalence of depression in China. A systematic review found that the prevalence of depressive symptoms in China ranged from 14.6 to 48.3% during the pandemic (42). Based on the estimated maximum incidence, we set *p* = 0.483. *q* = 1–*p, d* = absolute error or precision (when 10% < *p* <90%, it is suggested to set d as 0.05) (43, 44). According to the above value setting, the sample size calculated by the formula is 384 in our study. Considering that there will be invalid questionnaires in sample collection, we finally collected more than 384 samples. In total, 2,858 valid samples aged over 18 were obtained.

### Procedures

**Figure 1:**
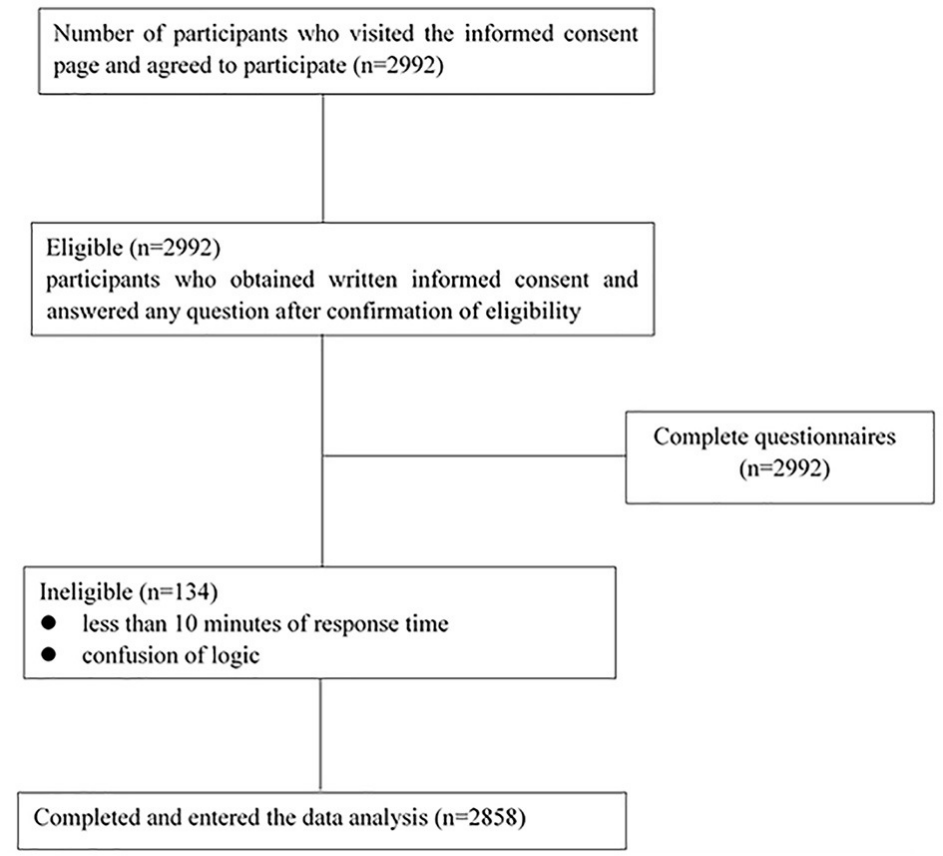
Flowchart of participant inclusion, exclusion, and completeness of surveys.

Participants were recruited through an online survey, and once participants accessed the site, they would visit the informed consent page. People agreed to participate by clicking the label of the informed consent page. The questionnaire usually took 10–20 min to complete, and participants were asked to answer each question during the process. After excluding 134 low-quality questionnaires (Exclusion criteria were any conditions that affected the quality of the questionnaire, including <10 min of response time, confusion of logic, etc.) total of 2,858 subjects was included in the final analysis. The flowchart of the study is shown in Figure 1.

### Measures

#### Dependent Variables

Depressive symptoms were assessed by the Center for Epidemiologic Studies Depression Scale (CEDS mainly used to evaluate the depressive symptoms of the general population in the previous week) (45). This scale has 20 items using 4 response options from 0 to 3, which has been found to have good reliability and validity in China (46, 47). The overall score ranged from 0 to 60. The higher scores indicate a high level of depressive symptoms. Based on the present data, the Cronbach's alpha of this scale was 0.93 in this study.

#### Independent Variables

In this study, care recipients mainly included the elderly and children. We assessed family care needs by asking the following question: “In the past 2 months, did they need to take care of elders.” The same questions were asked about childcare as well. The answer option was “Yes” or “No.” On this basis, we classified them into four types: care for the elderly only, care for the children only, care for both the elderly and the children, no need for care.

We measured family care needs satisfaction through the question: “In the past 2 months, whether the eldercare was satisfied.” The same questions were asked about childcare as well. The answer option was “Yes” or “No.” On this basis, we also classified them into four types: satisfy the elderly, satisfy the children, satisfy both the elderly and the children, and unsatisfied.

#### Moderator Variable

Economic stress was defined as economic hardships perceived by Chinese adults in the COVID-19 outbreak period. Based on related literature during the epidemic (6, 48), three items were developed to measure economic stress. It was assessed by the following items: economic loss, livelihood destruction, and lack of basic necessities. For each item, the options range from 1 (not at all) to 4 (very much). The overall score was summed by three items from 3 to 12. The higher scores refer to the heavier economic stress. Based on the present data, the Cronbach's alpha of this scale was 0.78.

#### Demographic and Socioeconomic Variables

Demographic and socioeconomic variables were as follows: gender (male/female), age (18–25, 26–30, 31–40, 41–50, 51, and over), ethnicity [Han (1)/else(0)], religion (believe/not believe), marriage (married/unmarried), party (yes/no), education (junior high school and below, high school/technical school, junior college, undergraduate, postgraduate, and above), occupations (medical workers, service staffs, social service workers, teachers and operators, students, workers and farmers, unemployed, and others), income (low/middle and high), living arrangement (live alone /live with spouses and children /live with parents and grandparents/else), Wuhan exposure (yes/no), media exposure [frequently (1)/sometimes (2)/less (3)/very less (4)] pre-psychological problems (yes/no), post-psychological problems (yes/no), 2-week illness (yes/no).

First, previous studies have found that females, younger people, unmarried persons, and non-Communist party members were prone to report higher depressive symptoms (49, 50). In addition, lower education levels and income were associated with high levels of depressive symptoms (49). At the same time, compared with other occupations, medical staff had higher depressive symptoms during the epidemic (51). Furthermore, another study found that pre-psychological problems, post-psychological problems, 2-week illness were significantly associated with higher depressive symptoms (52). Finally, Wuhan exposure, media exposure, ethnicity, religion, and living arrangement were also considered in the previous studies (52, 53). Therefore, these variables were included in this study because they had been examined in previous related studies (49–53).

Party was measured through the question: “Are you a member of the Communist Party of China?” with two response alternatives: “Yes” or “No.” Pre-psychological problems were based on this question “Did you have any psychological or emotional problems before the epidemic outbreak?” The answer option was “Yes” or “No.” Post-psychological problems were measured through the question: “Did you have any psychological or emotional problems after the epidemic outbreak” with two response alternatives: “Yes” or “No.” The 2-week illness was established through the question: “Have you had any headaches, fever, etc., in the last 2 weeks?” The answer option was “Yes” or “No.”

### Statistical Analysis

Descriptive statistics were conducted to analyze sample distributions. Means and standard deviations (SD) were used to describe continuous variables (depressive symptoms, economic stress), and frequency (*n*) and percentages (%) were used for categorical variables. Chi-square tests and *t*-test were used to test rural-urban differences between variables. Since dependent variables were continuous variables, several multiple linear regression (MLR) models were employed to examine the relationship between family care and depressive symptoms after adjusting for confounding variables. The interaction variables were created by economic stress and family care. Finally, several linear regression models were used to examine the interaction effects of economic stress on the relationship between family care and depressive symptoms. All analyses were conducted using Stata 15.0.

## Results

Table 1 shows the descriptive statistics of sample characteristics. Among 2,858 adult respondents, female (53.6%) and married (60.2%) accounted for over half, and the age distribution was mainly concentrated in the 31–40 years old range (31.2%). The level of education was mainly undergraduate (44.0%). The Han ethnicity (95.8%) and irreligious (90.5%) were more than 90%, and over 70% of respondents were not party members. 11.4% of respondents considered they were a low-income group. Most of the respondents were teachers and operators (22.7%), while social service workers and service staff each accounted for less than a tenth (8.0, 9.1%).

**Table 1:** Descriptive statistics of sample characteristics.

	**Total**		**Urban**		**Rural**		***P***
	***N***	**%**	***N***	**%**	***N***	**%**	
**Gender**							
Female	1,532	53.6	1,033	55.3	499	50.4	0.013
Male	1,326	46.4	835	44.7	491	49.6	
**Age**							
18–25	691	24.2	360	19.3	331	33.4	<0.001
26–30	645	22.6	394	21.1	251	25.4	
31–40	891	31.2	653	35.0	238	24.0	
41–50	400	14.0	324	17.3	76	7.7	
>51	231	8.1	137	7.3	94	9.5	
**Ethnicity**							
Else	120	4.2	81	4.3	39	3.9	0.615
Han	2,738	95.8	1,787	95.7	951	96.1	
**Religion**							
No	2,586	90.5	1,704	91.2	882	89.1	0.065
Yes	272	9.5	164	8.8	108	10.9	
**Party**							
No	2,132	74.6	1,278	68.4	854	86.3	<0.001
Yes	726	25.4	590	31.6	136	13.7	
**Marriage**							
Unmarried	1,137	39.8	638	34.2	499	50.4	<0.001
Married	1,721	60.2	1,230	65.8	491	49.6	
**Education**							
Junior high school and below	268	9.4	74	4.0	194	19.6	<0.001
High school/Technical school	387	13.5	196	10.5	191	19.3	
Junior College	488	17.1	289	15.5	199	20.1	
Undergraduate	1,257	44.0	954	51.1	303	30.6	
Postgraduate and above	458	16.0	355	19.0	103	10.4	
**Job**							
Medical workers	421	14.7	332	17.8	89	9.0	<0.001
Service staffs	259	9.1	173	9.3	86	8.7	
Social service workers	230	8.0	189	10.1	41	4.1	
Teachers and operators	648	22.7	461	24.7	187	18.9	
Students	424	14.8	221	11.8	203	20.5	
Workers and farmers	388	13.6	171	9.2	217	21.9	
Unemployed and others	488	17.1	321	17.2	167	16.9	
**Income**							
middle and high	2,531	88.6	1,734	92.8	797	80.5	<0.001
low	327	11.4	134	7.2	193	19.5	
**Living arrangement**							
Live alone	265	9.3	174	9.3	91	9.2	<0.001
Live with spouses and children	1,519	53.1	1,102	59.0	417	42.1	
Live with parents and grandparents	949	33.2	513	27.5	436	44.0	
Else	125	4.4	79	4.2	46	4.6	
**Wuhan exposure**							
No	2,445	85.5	1,608	86.1	837	84.5	<0.001
Yes	413	14.5	260	13.9	153	15.5	
**Media exposure**							
frequently	1,608	56.3	1,108	59.3	500	50.5	<0.001
sometimes	762	26.7	490	26.2	272	27.5	
less	259	9.1	153	8.2	106	10.7	
very less	229	8.0	117	6.3	112	11.3	
**Pre-psychological problems**							
No	2,440	85.4	1,589	85.1	851	86.0	0.519
Yes	418	14.6	279	14.9	139	14.0	
**Post-psychological problems**							
No	2,030	71.0	1,321	70.7	709	71.6	0.614
Yes	828	29.0	547	29.3	281	28.4	
**Two-week illness**							
No	2,657	93.0	1,734	92.8	923	93.2	0.686
Yes	201	7.0	134	7.2	67	6.8	
**Family care needs**							
Care for the elderly only	286	10.0	192	10.3	94	9.5	<0.001
Care for the children only	435	15.2	321	17.2	114	11.5	
Care for both the elderly and the children	335	11.7	251	13.4	84	8.5	
No need for care	1,802	63.1	1,104	59.1	698	70.5	
**Care needs satisfaction**							
Satisfy the elderly	241	22.8	162	21.2	79	27.1	0.237
Satisfy the children	397	37.6	294	38.5	103	35.3	
Satisfy both the elderly and children	196	18.6	146	19.1	50	17.1	
Unsatisfy	222	21.0	162	21.2	60	20.5	
	**Mean**	**SD**	**Mean**	**SD**	**Mean**	**SD**	
**Depressive symptoms**	14.51	11.68	14.18	11.57	15.12	11.88	0.043
**Economic stress**	7.78	2.54	7.67	2.55	8.0	2.49	0.001

*Chi-square tests and t-tests were used to report the significance of differences between urban sample and rural sample*.

In terms of care needs, the proportions of only caring for the elderly, caring for children, taking care of both the elderly and the children, and not needing caring were 10.0, 15.2, 11.7, and 63.1%, respectively. In terms of meeting care needs, the proportions of satisfying the elderly, satisfying the children, satisfying both the elderly and the children, and unsatisfied were 22.8, 37.6, 18.6, and 21.0%, respectively. The overall mean score was 14.51 (SD = 11.68; range 0–60) for the depressive symptoms and 7.78 (SD = 2.54; range 3–12) for the economic stress. There were significant differences in variables between urban and rural areas. For example, rural residents reported higher levels of depressive symptoms and economic stress than urban residents.

Table 2 presented the multiple linear regression analysis results for the relationship between family care needs and depressive symptoms. After controlling the relevant variables, sandwich-generation caregivers had a higher level of depressive symptoms than non-caregivers (*B* = 2.584, 95%CI: 1.254, 3.915). However, caring for the elderly or caring for the children was not significantly associated with depressive symptoms compared with non-caregivers. Economic stress was significantly associated with a higher level of depressive symptoms (*B* = 0.641, 95%CI: 0.481, 0.801). Besides, in urban areas, only sandwich-generation caregivers had a significant association with a higher level of depressive symptoms compared with non-caregivers (*B* = 2.193, 95%CI: 0.638, 3.748). While in rural areas, in addition to sandwich-generation caregivers, elderly caregivers also had a higher level of depressive symptoms than non-caregivers (*B* = 3.297, 95%CI: 0.688, 5.905; *B* = 3.135, 95%CI: 0.745, 5.525).

**Table 2:** Multiple liner regression analysis of the relationship between family care needs, economic stress and depressive symptoms.

	**Model 1-Overall**	**Model 2-Urban**	**Model 3-Rural**
	***B* (95% CI)**	***B* (95% CI)**	***B* (95% CI)**
Family care needs (Ref: No need for care)
Care for the elderly only	1.296 (−0.051, 2.643)	0.446 (−1.196, 2.087)	3.135^*^ (0.745, 5.525)
Care for the children only	1.134 (−0.094, 2.362)	0.949 (−0.510, 2.407)	1.508 (−0.823, 3.839)
Care for both the elderly and the children	2.584^***^ (1.254, 3.915)	2.193^**^ (0.638,3.748)	3.297^*^ (0.688, 5.905)
Economic stress	0.641^***^ (0.481, 0.801)	0.637^***^ (0.443,0.831)	0.626^***^ (0.340, 0.913)
Adjusted *R*-squared	0.168	0.171	0.164
*N*	2,858	1,868	990

* *p < 0.05*,

** *p < 0.01, and*

*** *p < 0.001*.

*B, the unstandardized coefficients. 95% confidence intervals in brackets. All confounding variables (gender, age, ethnicity, religion, party, marriage, education, job, income, living arrangement, Wuhan exposure, media exposure, pre-psychological problems, post-psychological problems, and 2-week illness) were controlled in the above models*.

Table 3 showed the multiple linear regression analysis results for the relationship between family care needs satisfaction and depressive symptoms. After controlling the relevant variables, the satisfactions of different types of family care needs were all significantly associated with lower levels of depressive symptoms compared with unmet care needs. These results also applied to the urban sample. While for rural areas, the results were not significant.

**Table 3:** Multiple liner regression analysis of the relationship between family care needs satisfaction, economic stress and depressive symptoms.

	**Model 1-Overall**	**Model 2-Urban**	**Model 3-Rural**
	***B* (95% CI)**	***B* (95% CI)**	***B* (95% CI)**
**Care needs satisfaction (Ref: Unsatisfy)**			
Satisfy the elderly	−3.915^***^ (−6.069, −1.762)	−5.133^***^ (−7.622, −2.644)	−1.203 (−5.693, 3.286)
Satisfy the children	−3.353^***^ (−5.258, −1.447)	−3.905^***^ (−6.068, −1.741)	−1.495 (−5.507, 2.517)
Satisfy both the elderly and children	−3.209^**^ (−5.381, −1.037)	−3.744^**^ (−6.219, −1.268)	−2.415 (−6.973, 2.143)
Economic stress	0.834^***^ (0.546, 1.122)	0.740^***^ (0.413, 1.068)	1.142^***^ (0.530, 1.754)
Adjusted R-squared	0.185	0.195	0.201
*N*	1,056	764	292

** *p < 0.01*,

*** *p < 0.001*.

*B, the unstandardized coefficients. 95% confidence intervals in brackets. All confounding variables (gender, age, ethnicity, religion, party, marriage, education, job, income, living arrangement, Wuhan exposure, media exposure, pre-psychological problems, post-psychological problems, and 2-week illness) were controlled in the above models. Note that, non-caregivers were not included in the family care satisfaction sample. Thus, only 1,056 samples were included in the model when considered the relationship between care needs satisfaction and depressive symptoms*.

**Figure 2:**
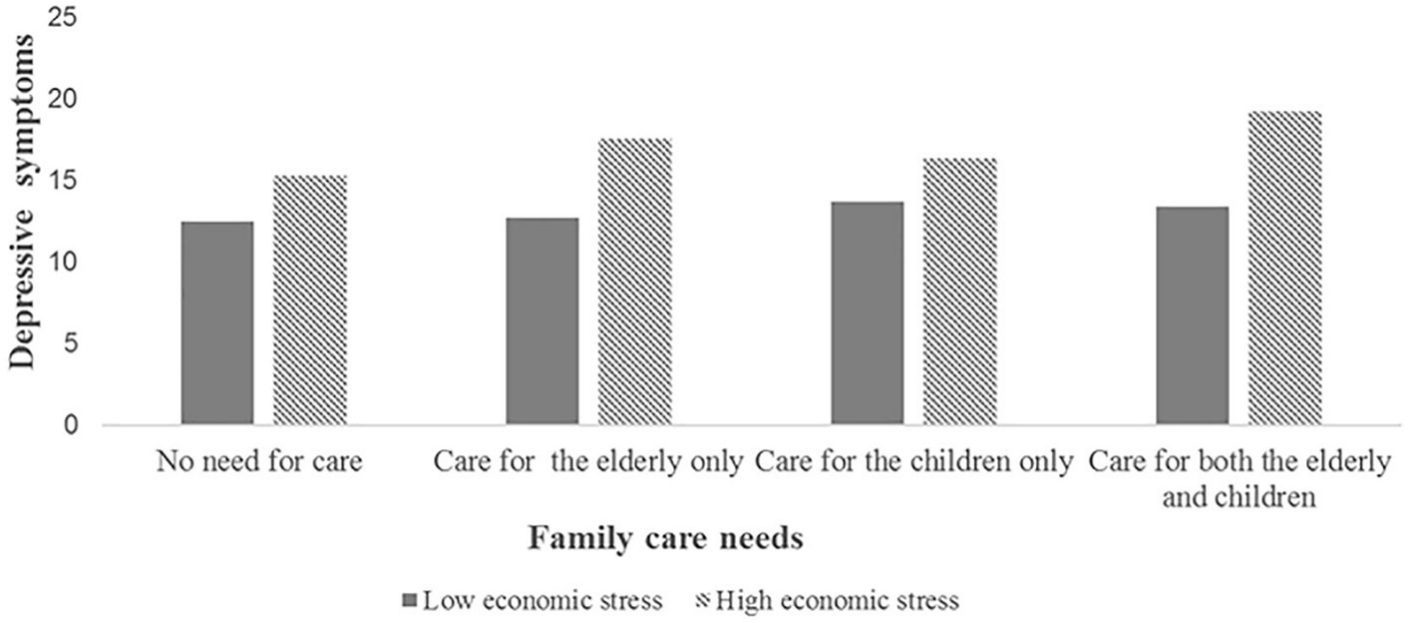
The interaction effect of family care needs and economic stress on depressive symptoms in the overall sample.

**Figure 3:**
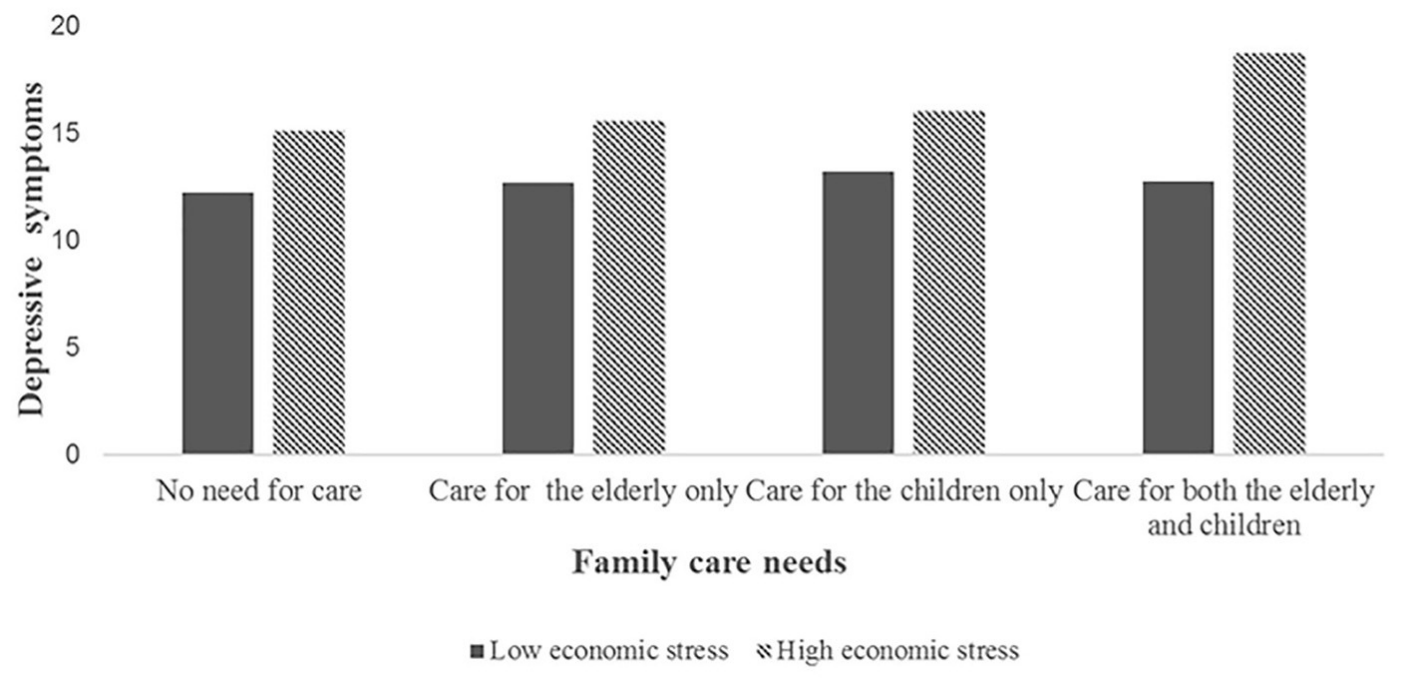
The interaction effect of family care needs and economic stress on depressive symptoms in the urban sample.

**Figure 4:**
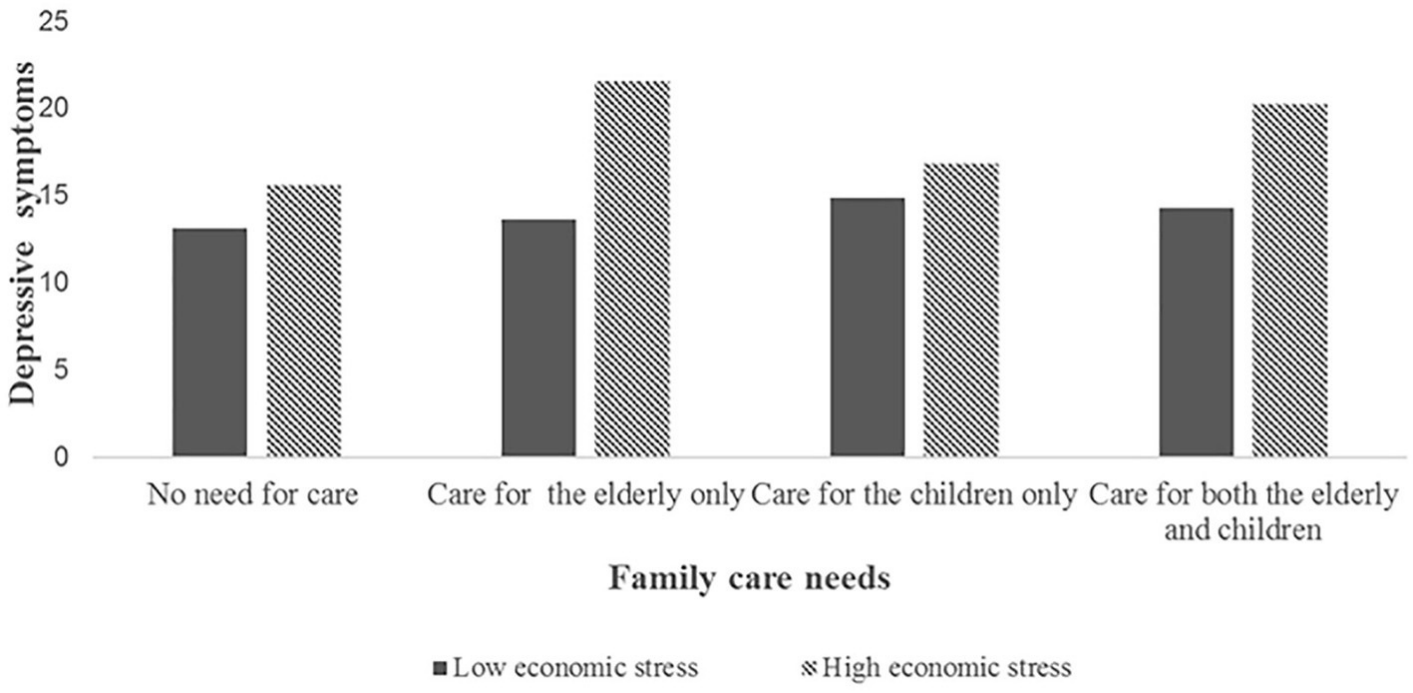
The interaction effect of family care needs and economic stress on depressive symptoms in the rural sample.

Table 4 and Figures 2–4 showed the interaction effect of family care needs and economic stress on depressive symptoms. The interaction effect of caring for both the elderly and the children and economic stress on depressive symptoms was significant (*B* = 0.605, 95%CI: 0.077, 1.134; Figure 2), and a similar result was also found in urban areas (*B* = 0.605, 95%CI: 0.004, 1.207; Figure 3). While in rural areas, economic stress only played a moderator role in the relationship between caring for the elderly and depressive symptoms (*B* = 1.106, 95%CI: 0.178, 2.035; Figure 4).

**Table 4:** Multiple liner regression analysis of the interaction effect of family care needs and economic stress on depressive symptoms.

	**Model 1-Overall**	**Mode2-Urban**	**Mode3-Rural**
	***B* (95% CI)**	***B* (95% CI)**	***B* (95% CI)**
**Family care needs (Ref: No need for care)**			
Care for the elderly only	1.268 (−0.079, 2.615)	0.468 (−1.174, 2.110)	2.965* (0.574, 5.355)
Care for the children only	1.122 (−0.105, 2.350)	0.948 (−0.510, 2.406)	1.485 (−0.845, 3.816)
Care for both the elderly and the children	2.404^***^ (1.064, 3.743)	2.128^**^ (0.572, 3.684)	2.724 (−0.098, 5.547)
Economic stress	0.551^***^ (0.356, 0.745)	0.571^***^ (0.328, 0.814)	0.490^**^ (0.160, 0.819)
**Family care needs** **×** **Economic stress (Ref: No need for care)**			
Care for the elderly only × Economic stress	0.407 (−0.139, 0.953)	−0.009 (−0.687, 0.670)	1.106^*^ (0.178, 2.035)
Care for the children only × Economic stress	–0.031 (−0.473, 0.411)	−0.026 (−0.540, 0.488)	−0.092 (−0.987, 0.804)
Care for both the elderly and the children × Economic stress	0.605^*^ (0.077, 1.134)	0.605^*^ (0.004, 1.207)	0.708 (−0.489, 1.905)
Adjusted R-squared	0.170	0.172	0.167
*N*	2,858	1,868	990

* *p < 0.05*,

** *p < 0.01*,

*** *p < 0.001*.

*B, the unstandardized coefficients. 95% confidence intervals in brackets. All confounding variables (gender, age, ethnicity, religion, party, marriage,education, job, income, living arrangement, Wuhan exposure, media exposure, pre-psychological problems, post-psychological problems, and 2-week illness) were controlled in the above models*.

Table 5 presented the interaction effect of care needs satisfaction and economic stress on depressive symptoms. The interaction effect of care needs satisfaction and economic stress on depressive symptoms was not observed significantly.

**Table 5:** Multiple liner regression analysis of the interaction effect of family care needs satisfaction and economic stress on depressive symptoms.

	**Model 1-Overall**	**Model 2-Urban**	**Model 3-Rural**
	***B* (95% CI)**	***B* (95% CI)**	***B* (95% CI)**
**Care needs satisfaction (Ref: Unsatisfy)**			
Satisfy the elderly	−3.784^***^ (−5.949, −1.619)	−4.893^***^ (−7.400, −2.386)	−1.589 (−6.113, 2.935)
Satisfy the children	−3.208^**^ (−5.126, −1.289)	−3.677^***^ (−5.854, −1.500)	−1.610 (−5.659, 2.439)
Satisfy both the elderly and children	−3.176^**^ (−5.365, −0.987)	−3.486^**^ (−5.975, −0.996)	−3.037 (−7.908, 1.834)
Economic stress	1.169^***^ (0.515, 1.822)	1.250^**^ (0.495, 2.005)	0.743 (−0.636, 2.121)
**Care needs satisfaction** **×** **Economic stress (Ref: Unsatisfy)**			
Satisfy the elderly × Economic stress	−0.196 (−1.058, 0.665)	−0.705 (−1.724, 0.315)	0.921 (−0.778, 2.619)
Satisfy the children × Economic stress	−0.747 (−1.528, 0.034)	−0.879 (−1.767, 0.009)	−0.048 (−1.700, 1.603)
Satisfy both the elderly and children × Economic stress	0.105 (−0.819, 1.029)	0.042 (−1.008, 1.092)	0.875 (−1.307, 3.058)
Adjusted R-squared	0.187	0.198	0.200
*N*	1,056	764	292

** *p < 0.01*,

*** *p < 0.001*,

*Bthe unstandardized coefficients. 95% confidence intervals in brackets. All confounding variables (gender, age, ethnicity, religion, party, marriage, education, job, income, living arrangement, Wuhan exposure, media exposure, pre-psychological problems, post-psychological problems, and 2-week illness) were controlled in the above models*.

## Discussion

In this study, family care was partially associated with depressive symptoms. Besides, economic stress strengthened the association between family care needs and depressive symptoms. However, the moderating effects of economic stress were only found in sandwich-generation caregivers and rural elderly caregivers. Furthermore, this study also found that the correlation between family care and depressive symptoms was different between urban and rural areas. Some hypotheses in this study have been confirmed, which may provide a theoretical basis for the formulation of mental health intervention strategies for family caregivers.

This study revealed that in comparison with the non-caregivers, the level of depressive symptoms among sandwich-generation caregivers was higher. Similar results were found in previous findings (54). According to the family-role overload theory, family caregivers played multiple roles and did not have enough time and energy to cope with the difficult situations, making them vulnerable to stress (55, 56). Sandwich-generation caregivers have to continue to provide not only complex care to the old and the children but also need to cope with increasingly difficult work and care problems at home during crisis (3), which put them at a higher risk for depressive symptoms. Furthermore, the multi-generational family structure may become an essential source of transmission. For example, older people and children may be at high risk of infection because of sandwich-generation caregivers who work outside the home (29). Consequently, to protect the care recipients, sandwiched caregivers will worry more about being infected. In addition, the social disruption caused by COVID-19 affected the quality of family relationships (6). Not surprisingly, the family relationship of the sandwiched caregivers will be further strained. Therefore, the mental health of the sandwich-generation caregivers should be concerned during the epidemic.

However, the relationship between family care needs and depressive symptoms showed urban-rural differences. We found that caring for the elderly only was related to the high levels of depressive symptoms in rural areas, besides caring for both the elderly and children. The results can be explained as follows: First, from age structure, the aging degree of the rural population has exceeded that of the urban population, presenting the phenomenon of urban-rural aging population reversed (57). Second, due to rural labor out-migration, elderly care problems were more severe in rural area (58). Finally, the COVID-19 has posed greater challenges for finite healthcare resources in rural regions (e.g., shortages of resources and staff), especially for the elderly with severe illness and chronic diseases (59). Given these differences, rural caregivers face considerable barriers to caring for the elderly in this harsh period. As a result, it is unsurprising to see the relatively high levels of depressive symptoms among rural elderly caregivers. The findings of this study remind us to pay attention to the mental health problems of sandwich-generation caregivers and rural elderly caregivers during the epidemic. On the one hand, in the intervention policies formulation process, we should be aware that sandwich-generation caregivers face a two-fold vulnerability: high risk of infection and psychological crisis. On the other hand, more policies are needed to address the care dilemmas faced by rural elderly caregivers during the crisis.

Furthermore, our results showed that care needs satisfaction was correlated with less depressive symptoms compared with unmet care needs. This was contrary to the results of a previous study which found that meeting the continuous care needs of family members was a stress burden that increased the risk of depressive symptoms among caregivers (60). According to Maslow's hierarchy of needs theory, there was a strong link between the satisfaction of psychological needs and mental health. Although family care was an unpaid activity, caregivers also experienced satisfaction in the process of meeting care recipients' needs (61). However, this relationship was not pronounced in rural areas. The reason for this might be that as a result of the rural labor out-migration rural care recipients were inherently difficult to obtain family care (33). Even worse, COVID-19 posed new risks to the satisfaction of basic living needs, in rural neighborhoods. For example, getting health care and seeking family assistance were all accompanied by challenges (59). Thus, some rural family care needs may not be adequately satisfied in this particular period. Based on this finding, effective measures are needed to meet the family care needs of COVID-19. In particular, we should provide timely psychological counseling to the caregivers who do not satisfy the family care needs and affirm their efforts in this challenging period (3).

Moreover, economic stress played a moderator role in the relationship between caring for both the elderly and children and depressive symptoms. Sandwich-generation caregivers with higher economic stress were at increased risk of depressive symptoms. This is consonant with previous research that shows that compared with other types of care, sandwich-generation caregivers tended to consume more resources, including economic resources, which posed an increased risk of depression (54). This interaction may be explained in line with COR theory (conservation of resources) that stated that individuals tended to preserve, protect, and build resources. Therefore, the potential or actual loss of resources would make them tense and stressed (62). Sandwich-generation caregivers needed to pay not only for the medical resources and services of the elderly but had to bear the education expenses of the children also (63). Consequently, they might experience a greater loss of resources than other types of caregivers. Especially, sandwich-generation caregivers who had greater economic stress would have fewer resources to cope with caregiving stressors during the COVID-19 epidemic, which may be detrimental to their mental health.

It is interesting to note that the moderating role of economic stress on the association between family care needs and depressive symptoms was established only for elderly caregivers in rural areas. Two channels may explain this result. On the one hand, compared with the urban elderly, most rural elderly did not have a pension and mainly relied on their family members (64), so they had fewer economic resources to cushion financial losses caused by COVID-19 (59). On the other hand, rural elderly caregivers had less income to help them cope with the financial risks of COVID-19 compared with their counterparts in urban regions. Restrictive policies related to the epidemic caused many rural laborers to fail to obtain jobs, resulting in a decline in income levels (40). Meanwhile, because of the urban-biased social security policy, many migrant workers could not get financial support from unemployment insurance in this pandemic (65). Thus, focusing on urban-rural differences, it is imperative to establish effective policies to provide financial support for rural elderly caregivers.

## Limitation and Implications

This study has the following limitations. First, the cross-sectional data cannot be causally inferred. More longitudinal studies are needed to establish causal associations between family care and depressive symptoms. Secondly, some potential confounders were not controlled in the analysis, such as COVID-19 quarantine, and change in lifestyle due to quarantine. Therefore, these need to be considered in future studies. Thirdly, the data was collected online and based on non-random sampling, possibly leading to the risk of selection bias. In addition, we only measured whether the elderly and children needed care, without specifying how many hours of care. Furthermore, we did not test the degree of care needs satisfaction. Therefore, further research is required to examine them. Next, because our data were collected at the early stage of the COVID-19 outbreak, it was not clear whether the relationship between family care and depressive symptoms would sustain in the long run. Thus, long-term and larger-scale region studies are needed to further test our conclusions. Finally, the scales used in our study are self-reporting and screening tools, not clinical diagnostic instruments. To improve the accuracy of the research, more studies with diagnostic criteria are needed in the future.

Despite these limitations, this study provided some implications. Firstly, this study found that family care needs were associated with a high level of depressive symptoms among Chinese adults, especially sandwich-generation caregivers. Thus, psycho-educational intervention should be delivered for family caregivers. Secondly, it is essential to meet the care needs of family care. Therefore, timely information should be collected from families with care needs, including care burden and specific care needs. Furthermore, given the economic stress exacerbating sandwich-generation caregivers' depressive symptoms, a comprehensive policy should be established to provide economic support (livelihood support, basic material support, and economic subsidy) for the sandwich-generation caregivers. Finally, considering the differences in family care between urban and rural areas, we should give more psychological intervention and financial support to urban sandwich-generation caregivers and rural elderly caregivers.

## Conclusion

The study suggests family care is partially associated with depressive symptoms among Chinese adults during the COVID-19 outbreak. Moreover, the moderating effects of economic stress are only found in sandwich-generation caregivers and rural elderly caregivers. Therefore, we advocate psychological intervention for the family caregivers' mental health. In addition, more effective policies should be developed to provide financial support for family caregivers, especially for sandwich-generation caregivers and rural elderly caregivers.

## Data Availability Statement

The datasets used and/or analyzed during the current study are available from the corresponding author on reasonable request.

## Ethics Statement

The studies involving human participants were reviewed and approved by Peking University. The patients/participants provided their written informed consent to participate in this study. Written informed consent was obtained from the individual(s) for the publication of any potentially identifiable images or data included in this article.

## Author Contributions

LZ, JG, and CL drafted the manuscript. FA, MS, XW, YW, and JW were involved in revising the manuscript. All authors were involved in writing the manuscript and approve of its final version.

## Conflict of Interest

The authors declare that the research was conducted in the absence of any commercial or financial relationships that could be construed as a potential conflict of interest.
